# Melioidosis and Hairy Cell Leukemia in 2 Travelers Returning from Thailand

**DOI:** 10.3201/eid1903.121329

**Published:** 2013-03

**Authors:** Benjamin Rossi, Loïc Epelboin, Stéphane Jauréguiberry, Maryline Lecso, Damien Roos-Weil, Jean Gabarre, Philippe A. Grenier, François Bricaire, Eric Caumes

**Affiliations:** Author affiliations: Groupe Hospitalier Pitié-Salpêtrière, Paris, France (B. Rossi, L. Epelboin, S. Jauréguiberry, M. Lecso, D. Roos-Weil, J. Gabarre, P. Grenier, F. Bricaire, E. Caumes);; Université Pierre et Marie Curie–Paris VI, Paris (L. Epelboin, S. Jauréguiberry, D. Roos-Weil, P.A. Grenier, F. Bricaire, E. Caumes)

**Keywords:** melioidosis, Burkholderia pseudomallei, bacteria, hairy cell leukemia, travel medicine, Thailand

**To the Editor:** Patients with underlying medical conditions travel more than ever (*1*), and such travelers may be exposed to uncommon infections (*2*). We report 2 cases of melioidosis and hairy cell leukemia in travelers returning from Thailand.

Case-patient 1 was a 48-year-old man hospitalized in Paris with fever, asthenia, chills, and pancytopenia after returning from a 1-week visit to Thailand where he had been in flooded regions (Koh Samui and Koh Samet). Clinical examination showed a temperature of 40°C and mucocutaneous pallor. Laboratory tests showed a hemoglobin level of 7.9 g/dL, a platelet count of 33 × 10^9^/L, a leukocyte count of 1.3 × 10^9^ cells/L, a polymorphonuclear cell count of 0.77 × 10^9^ cells/L, a monocyte count of 0, and a C-reactive protein level of 158 mg/L. Results of tests for HIV, dengue, and malaria were negative.

Presumptive antimicrobial drug treatment with piperacillin/tazobactam (12 g/1.5 g/d) was initiated at admission. A blood smear showed 10% hairy cells, and a bone marrow biopsy confirmed a diagnosis of hairy cell leukemia and interstitial infiltration of CD20-positive, monoclonal antibody DBA.44–positive, and tartrate-resistant acid phosphatase–positive cells.

Because of persistent unexplained fever, full-body computed tomography (CT) was performed and showed multiple liver, spleen, and lung abscesses (Figure, panels A and B). Culture of a CT scan–guided liver abscess puncture specimen was positive for *Bukholderia pseudomallei* after 12 days of antimicrobial drug treatment. Treatment was changed to ceftazidime (120 mg/kg/d) and trimethoprim/sulfamethoxazole (TMP/SMX) (10/50 mg/kg/d), and oral doxycycline (200 m/d) for 3 weeks. The outcome was good.

Oral treatment with TMP/SMX and doxycycline (200 mg/d) was continued for 20 weeks. Treatment for hairy cell leukemia with cladribine was initiated after 10 weeks of antimicrobial drug treatment. Two years later, the patient showed complete remission of hairy cell leukemia and melioidosis.

Case-patient 2 was a 64-year-old man hospitalized in Paris for persistent fever 16 days after his return from Thailand. Two months earlier in Thailand, he had received treatment for hepatosplenic melioidosis with ceftazidime (120 mg/kg/d), TMP/SMX (10/50 mg/kg/d), and doxycycline (200 mg/d) for 15 days, and then oral amoxicillin/clavulanic acid (3 g/d) for 3 months. At admission, he had fever, chills, abdominal pain, and cough. Clinical examination showed a temperature of 40°C and left lung crackles. Chest and abdomen CT images showed a focus of lung consolidations (Figure, panels C and D), left pleural effusion, pericarditis, and spleen abscesses. Laboratory tests showed a leukocyte count of 1.05 × 10^9^ cells/L, a monocyte count of 0.04 × 10^9^ cells/L, a hemoglobin level of 7.9 g/dL, a platelet count of 62 × 10^9^/L, and a serum ferritin level of 8,530 IU/L.

Blood cultures were positive for *B. pseudomallei*. The strain was sensitive to amoxicillin/clavulanic acid. Bone marrow aspiration and biopsy showed hemophagocytosis and interstitial infiltration of CD20-positive, monoclonal antibody DBA.44–positive, CD 103-positive, CD25-positive, CD11c-positive, and CD123-positive cells, leading to a diagnosis of hairy cell leukemia. The patient was given a 2-week course of intravenous TMP/SMX (10 mg/50 mg/kg/d), oral doxycycline (200 mg/d), and intravenous ceftazidime (120 mg/kg/d), followed by a 6-month course of oral TMP/SMX (50 mg/10 mg/kg/d) and doxycycline (4 mg/kg/d). The condition of the patient improved and pancytopenia resolved. Thus, he did not require any treatment for hairy cell leukemia. No relapse of melioidosis occurred.

Melioidosis is endemic to the Pacific region and Southeast Asia (*3*,*4*). Most cases reported in other regions are imported (*5*). In Thailand, where both patients had traveled, the number of cases increased from 11.5/100,000 inhabitants in 1997 to 21.3/100,000 in 2006 (*6*). The 2 main routes of transmission are transcutaneous and aerosols. Natural disasters, such as flooding, are a risk factor for melioidosis, as for case-patient 1.

This disease has an overall mortality rate of 50%. The clinical spectrum ranges from acute septicemia (mortality rate 80%) to the subacute form. *B. pseudomallei* is difficult to detect by culture of biologic samples, and serologic analysis or PCR for this bacteria are not routinely available. Therefore, a diagnosis of melioidosis can be easily missed.

Melioidosis occurs mainly in patients with underlying diseases such as diabetes (37%–60% of cases), chronic alcoholism (12%–39%), thalassemia, and chronic nephropathy, and in persons receiving long-term corticosteroid treatment (*7*). Reports of patients with melioidosis and hematologic malignancies or solid cancers are scarce (*4*,*5*,*7*). Hairy cell leukemia could now be included in this group of diseases.

Hairy cell leukemia is a rare chronic B-cell lymphoproliferative disorder characterized by pancytopenia; splenomegaly; and infiltration of the bone marrow, spleen, and liver by malignant B cells that have hair-like cytoplasmic projections (*8*,*9*). The incidence of hairy cell leukemia is <1 case/100,000 population/year, and the disease accounts for ≈2%–3% of all leukemias in adults in the United States (*8*). Infections are a common complication for patients with this disease (*10*).

These 2 cases of imported melioidosis show that travelers with hematologic malignancies are at risk for such infections (*1*). Immunocompromised travelers might be first sentinels for ongoing endemic diseases. When travelers return with uncommon diseases, physicians should check for underlying diseases. Physicians providing care for patients with hairy cell leukemia should be aware of the risk for contracting melioidosis.

**Figure:**
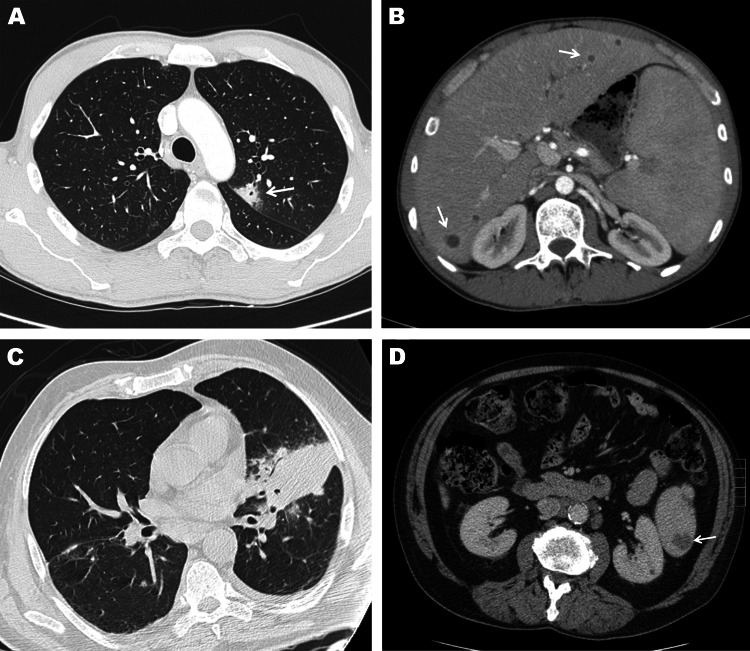
Computed tomography (CT) images of the chest and abdomen of case-patient 1 showing A) a subpleural nodular and cavitary lesion (arrow) in the left upper lobe of the lung and B) multiple small round liver abscesses, seen as multiple foci of ill-defined areas of hypoattenuation (arrows), and enlargement of the spleen. CT images of the chest and abdomen of case-patient 2 showing C) a focal area of parenchymal consolidation in the left lung associated with an ipsilateral mild pleural effusion and D) and a spleen abscess (arrow).

## References

[1] Wieten RW, Leenstra T, Goorhuis A, van Vugt M, Grobusch MP. Health risks of travelers with medical conditions: a retrospective analysis. J Travel Med. 2012;19:104–10 and. 10.1111/j.1708-8305.2011.00594.x 22414035

[2] McCarthy AE, Mileno MD. Prevention and treatment of travel-related infections in compromised hosts. Curr Opin Infect Dis. 2006;19:450–5 and. 10.1097/01.qco.0000244050.15888.6f 16940868

[3] Cheng AC, Currie BJ. Melioidosis: epidemiology, pathophysiology, and management. Clin Microbiol Rev. 2005;18:383–416 and. 10.1128/CMR.18.2.383-416.2005 15831829 PMC1082802

[4] White NJ. Melioidosis. Lancet. 2003;361:1715–22 and. 10.1016/S0140-6736(03)13374-0 12767750

[5] Cahn A, Koslowsky B, Nir-Paz R, Temper V, Hiller N, Karlinsky A, Imported melioidosis, Israel, 2008. Emerg Infect Dis. 2009;15:1809–11 and. 10.3201/eid1511.090038 19891871 PMC2857218

[6] Limmathurotsakul D, Wongratanacheewin S, Teerawattanasook N, Wongsuvan G, Chaisuksant S, Chetchotisakd P, Increasing incidence of human melioidosis in northeast Thailand. Am J Trop Med Hyg. 2010;82:1113–7 and. 10.4269/ajtmh.2010.10-0038 20519609 PMC2877420

[7] Salam AP, Khan N, Malnick H, Kenna DT, Dance DA, Klein JL. Melioidosis acquired by traveler to Nigeria. Emerg Infect Dis. 2011;17:1296–8 and. 10.3201/eid1707.110502 21762592 PMC3381395

[8] Goodman GR, Bethel KJ, Saven A. Hairy cell leukemia: an update. Curr Opin Hematol. 2003;10:258–66 and. 10.1097/00062752-200307000-00002 12799530

[9] Bouroncle BA, Wiseman BK, Doan CA. Leukemic reticuloendotheliosis. Blood. 1958;13:609–30 .13560561

[10] Kraut E. Infectious complications in hairy cell leukemia. Leuk Lymphoma. 2011;52(Suppl 2):50–2 and. 10.3109/10428194.2011.570819 21504285

